# The pharmacokinetics and pharmacodynamics of danirixin (GSK1325756) − a selective CXCR2 antagonist − in healthy adult subjects

**DOI:** 10.1186/s40360-015-0017-x

**Published:** 2015-06-20

**Authors:** Bruce E. Miller, Sunil Mistry, Kevin Smart, Paul Connolly, Donald C. Carpenter, Hiran Cooray, Jackie C. Bloomer, Ruth Tal-Singer, Aili L. Lazaar

**Affiliations:** Clinical Discovery, Respiratory Therapy Area Unit, GlaxoSmithKline R&D, 709 Swedeland Road, King of Prussia, PA, 19406 USA; Clinical Statistics, GlaxoSmithKline R&D, Stockley Park West, Uxbridge, Middlesex, UB11 1BT UK; Clinical Pharmacology Modeling and Simulation, GlaxoSmithKline, London, UK; Product Development, Platform Technology and Science, GlaxoSmithKline R&D, Park Road, Ware, Hertfordshire, SG 12 0DP UK; Respiratory Therapy Area Unit, GlaxoSmithKline R&D, 709 Swedeland Road, King of Prussia, PA, 19406 USA; Clinical Pharmacology Sciences and Study Operations, GlaxoSmithKline, London, UK; Drug Metabolism and Pharmacokinetics, GlaxoSmithKline R&D, Park Road, Ware, Hertfordshire, SG 12 0DP UK; Discovery Medicine, Respiratory Therapy Area Unit, GlaxoSmithKline R&D, 709 Swedeland Road, King of Prussia, PA, 19406 USA; Roche Pharmaceuticals, Welwyn Garden City, UK; Global Development, Amgen Inc., Horsham, West Sussex, UK

**Keywords:** CXCR2 antagonist, Danirixin, GSK1325756, Chronic obstructive pulmonary disease, Pharmacokinetics, Pharmacodynamics, Omeprazole

## Abstract

**Background:**

Excessive neutrophil presence and activation is important in a number of acute and chronic inflammatory diseases. The CXCR2 chemokine receptor is important in controlling the extravasation and activation of neutrophils. Selective antagonism of the CXCR2 receptor is a potential approach to reducing neutrophil migration and activation. Danirixin, is a small molecule, CXCR2 antagonist being evaluated as a potential anti-inflammatory medicine.

**Methods:**

(1) First time in human (FTIH) double-blind, placebo-controlled study to evaluate the safety, pharmacokinetics, and pharmacodynamics of single ascending and repeat oral doses of danirixin in healthy male subjects; (2) single-dose study of age, gender, food, and proton-pump inhibitor effects on the pharmacokinetics of danirixin in healthy adult subjects; and placebo-controlled study of the pharmacokinetics of danirixin in healthy elderly subjects.

**Results:**

There were no serious adverse events and no adverse events considered to be of clinical relevance. There were no withdrawals due to adverse events. Systemic exposure following single doses of danirixin 10 mg, 25 mg, 50 mg, 100 mg, and 200 mg increased with increasing dose. Engagement of pharmacology was demonstrated as inhibition of ex-vivo CXCL1-induced CD11b expression on peripheral blood neutrophils when compared to placebo (approximately 50 % for 50 mg and 100 mg danirixin, and 72 % at 200 mg). There was a 37 % decrease in Cmax and a 16 % decrease in AUC (0-∞) following administration of danirixin in the presence of food. Cmax also decreased by 65 % when danirixin 100 mg was administered following omeprazole 40 mg once daily for 5 days. The AUC (0-∞) and Cmax were 50 % lower in elderly subjects compared with younger subjects.

**Conclusion:**

The dose-dependent inhibition of agonist-induced neutrophil activation following single and repeated once daily oral administration of danirixin suggests that this CXCR2 antagonist may have benefit in neutrophil-predominant inflammatory diseases. Co-administration with food, gastric acid reducing agents, and variable exposure in the elderly have important clinical implications that need to be taken into consideration in subsequent clinical evaluations.

**Trial registration:**

ClinicalTrials.gov identifiers: NCT01209052 and NCT01209104

## Background

Chemokines acting through specific receptors are able to recruit leukocytes to sites of inflammation, resulting in the subsequent release of a number of mediators, including neutrophil elastase and matrix metalloproteinases, which likely play an important role in disease pathophysiology [1]. For several acute and chronic pulmonary diseases, chemokines are thought to be important mediators driving the tissue destruction and airway and parenchymal remodeling associated with disease progression [2, 3].

Selective antagonism of the interaction between the CXCR2 chemokine receptor and its various ligands provides a potential targeted strategy for reducing the underlying inflammation that contributes to the deleterious effects of an excessive neutrophil response [2]. In particular, a CXCR2 antagonist may be useful in the treatment of respiratory diseases such as chronic obstructive pulmonary disease (COPD) that are associated with a high tissue burden of activated neutrophils. The potential for CXCR2 antagonism to reduce neutrophil recruitment into the lung has been demonstrated previously in experimental human models of lung inflammation (ozone challenge) [4, 5] and in initial studies in patients with severe asthma and COPD [6–8]. Transient reductions in blood neutrophils have been observed with two recent small molecule CXCR2 antagonists following short and long term oral administration leading to concerns about a potential impact on host defense and innate immunity, particularly with chronic use. It is therefore desirable to identify a CXCR2 antagonist that would have the intended efficacy in the target tissues without substantially impacting circulating neutrophil counts.

Danirixin is a small molecule, non-peptide, high affinity (IC_50_ for CXCL8 binding = 12.5 nM), selective, and reversible CXCR2 antagonist. This compound has demonstrated potent antagonism of CXCR2 activity in vitro and anti-inflammatory effects in various preclinical models. Its pharmacologic activity and duration of action following oral administration support its potential use as an oral, anti-inflammatory agent.

The results of two clinical pharmacology studies with danirixin are described in this report. The first time in human (FTIH) study was conducted to investigate the safety, tolerability, pharmacokinetics, and pharmacodynamics of single and repeat oral doses of danirixin in healthy adult male subjects. The second study explored the effects of age, gender, food (high fat meal), and a proton-pump inhibitor (omeprazole) on the pharmacokinetics of danirixin.

## Methods

### Danirixin formulation

**Fig. 1:**
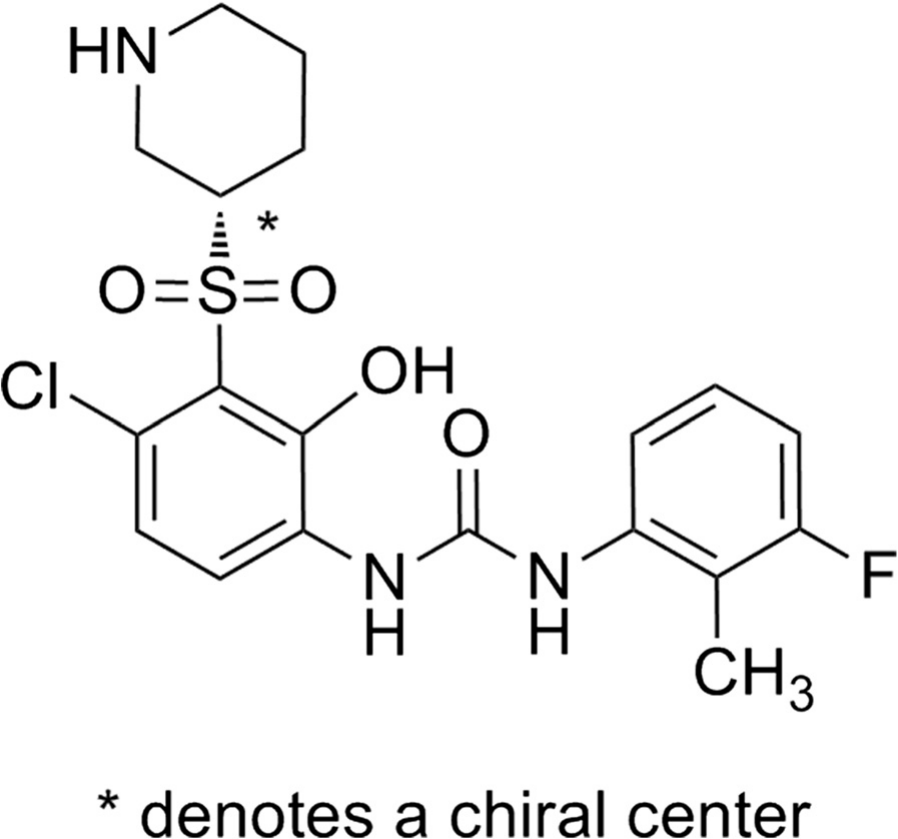
Danirixin Free Base

The structure of danirixin free base is shown in Fig. 1. Immediate release (IR) tablets contained danirixin free base and standard excipients including mannitol, microcrystalline cellulose, HPMC, croscarmellose sodium and magnesium stearate.

### Study designs and study subjects

Clinical studies were conducted in accordance with the Declaration of Helskini. Prior to study initiation approvals from appropriate regulatory authorities and ethics committee/institutional review boards were obtained. Written, informed consent was obtained from participating subjects prior to the performance of any study related assessments and procedures.

Study 1, FTIH (GSK Protocol CX3112483, ClinicalTrials.gov identifier NCT01209052, reviewed and approved by the Brent Medical Ethics Committee, Harrow, UK)

**Fig. 2:**
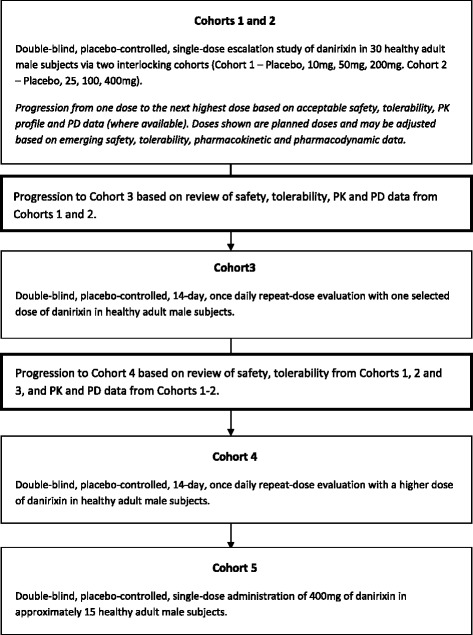
Study design algorithm for Study 1 (FTIH)

The first time in human study was a single-center, randomized, double-blind, placebo-controlled, adaptive design comprising five cohorts. Two cohorts of 15 healthy non-smoking male subjects aged 18 to 65 years were randomized to receive single escalating oral doses of danirixin. Cohort 1 received doses of 10 mg, 50 mg and 200 mg; Cohort 2 received doses of 25 mg, 100 mg (as a single tablet) and 100 mg (as 2 × 50 mg tablets), as well as placebo. An interlocking design allowed a period of at least 7 days to elapse between dosing in one cohort and administration of a higher dose in the other cohort. Subjects in Cohorts 3 and 4 received repeat doses of danirixin or placebo once daily for 14 days in a parallel-group design. Fourteen subjects in each cohort were randomized 5:2 to receive danirixin 50 mg (Cohort 3) or 200 mg (Cohort 4) or placebo. Cohort 5 consisted of 15 subjects. Ten subjects were randomized to receive danirixin 400 mg and five subjects received placebo as a single dose. The schematic for the study design of the FTIH study is depicted in Fig. 2.

Study 2, Fed versus Fasted, with Omeprazole (GSK Protocol CX3113722, ClinicalTrials.gov identifier NCT01209104, reviewed and approved by the Western Institutional Review Board, Olympia, WA, USA)

**Fig. 3:**
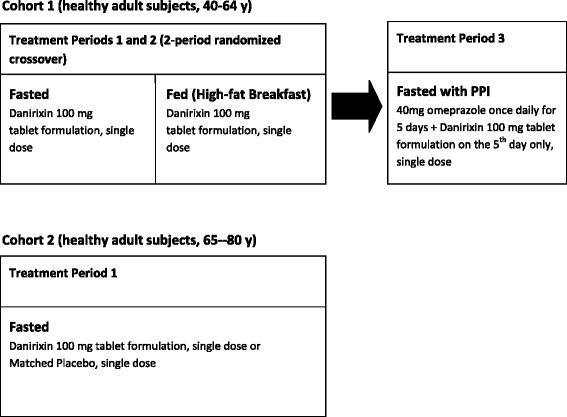
Study design algorithm for Study 2 (Fed versus Fasted, Elderly, Omeprazole Interaction)

Study 2 was conducted at a single center in two parallel parts as depicted in Fig. 3. Cohort 1 was a partially randomized, open-label, single-dose, crossover study in 16 healthy male and female adult subjects between 40 and 64 years of age. In treatment periods 1 and 2, subjects were randomized to two single dose treatment regimens in a crossover fashion, in the fed (after a high-fat meal) and fasted states. In treatment period 3, subjects were administered omeprazole once daily for 5 days. On the fifth day, all subjects received danirixin 100 mg together with the final dose of omeprazole.

Cohort 2 was a single-dose, double-blind, parallel group investigation in 16 healthy elderly individuals between 65 and 80 years of age. Subjects were randomized 3:1 to receive danirixin 100 mg or placebo in the fasting state.

### Safety

For each subject, adverse events and serious adverse events were collected from the start of first dosing until the final follow-up contact. Safety was monitored by the measurement of ECGs, vital signs, clinical laboratory assessments (clinical chemistry, hematology, and urinalysis), and assessment of adverse events.

### Determination of danirixin concentrations in whole blood and pharmacokinetic assessment

Whole blood concentrations of danirixin free base were determined using a validated analytical method based on extraction from a dried blood spot [9], with a 3 mm disc being punched from a 0.015 mL sample on Whatman FTA™ cards. Danirixin was extracted using methanol (0.1 mL) containing isotopically labeled [^2^H7]-danirixin (racemic version of danirixin) at a concentration of 50 ng/mL as an internal standard. The extraction tubes were shaken for 1 h at ambient temperature, prior to the supernatant being transferred to clean tubes. The supernatant (5 μL) was injected onto a high-performance liquid chromatography system utilizing a Thermo Scientific Hypersil Gold C18 (5 μm packing 50 × 4.6 mm) column (supplied by Thermo Scientific, Hempstead, UK), eluted using an isocratic composition: 55:45 (v/v) of aqueous 10 mM ammonium formate containing 0.1 % formic acid (A) and acetonitrile (B). Danirixin free base has a retention time of approximately 1 min and was detected using tandem mass spectrometry on a Sciex 5000 (Applied Biosystems, Warrington, UK) using Turbolon Spray in positive polarity mode. Mass transitions monitored were 84 and 89 from precursor ions of 442 and 449 for danirixin and the internal standard. The assay had a linear dynamic range of 5 – 1000 ng/mL and quantification was performed using peak area ratios with 1/×^2^ weighted linear regression. Assay quality control samples were 15 ng/mL (low), 200 ng/mL (medium) and 800 ng/mL (high). The average within-run precision (%CV) was 9.9, 7.2 and 9.0 respectively, for the low, mid and high quality controls for study CX3112483 and 18.9, 5.9 and 5.2, respectively, for the low, mid and high quality controls for study CX3113722.

Pharmacokinetic parameters were determined from the blood concentration-time data for danirixin. The pharmacokinetic parameters were calculated based on actual sampling times using standard non-compartmental analysis in WinNonlin Professional, Version 5.2. The area under the blood concentration-time curve (AUC) was determined from the time of dosing to the last quantifiable concentration (AUC_(0-t)_) using the lin – log trapezoidal rule. The apparent terminal elimination half-life (t_1/2_) was obtained as the ratio of ln2/λ_z_, where λ_z_ is the terminal phase rate constant, estimated by linear regression through the log transformed terminal data. AUC_(0-t)_ was extrapolated to infinity (AUC_(0-∞)_) as the sum of AUC_(0-t)_ and C_t/_λ_z_, where C_t_ is the last observed blood concentration and λ_z_ is the terminal phase rate constant.

### Flow cytometric analysis of CD11b expression on blood neutrophils

CXCR2 ligand-induced cell surface expression of CD11b has been demonstrated to be a useful pharmacodynamic biomarker for the effects of CXCR2 antagonists [4]. To assess the pharmacodynamic effects of danirixin following oral administration, CXCL1-induced CD11b cell surface expression on blood neutrophils was determined with a whole blood assay as previously described [4, 10].

### Statistical analyses

#### Study 1

The primary endpoint was safety and tolerability assessed by clinical monitoring of blood pressure, pulse rate, ECG, and clinical laboratory assessments as well as reporting of adverse events. Secondary endpoints included: 1) AUC (0-∞), AUC (0-t), maximum blood concentration (Cmax), time to maximum blood concentration (Tmax), and terminal half-life (t_1/2_). 2) *ex vivo* CXCL1-induced CD11b cell surface expression on peripheral blood neutrophils, and 3) the relationship between the blood concentration of danirixin and *ex vivo* CXCL-1-induced CD11b cell surface expression on peripheral blood neutrophils.

In the single dose cohorts, dose proportionality was calculated on AUC (0 to ∞) and Cmax for Cohorts 1 and 2 and repeated again with Cohorts 1, 2, and 5. The power model analysis was performed on log_e_-transformed AUC (0 to ∞) and Cmax for danirixin. For each of these parameters, a mixed effects model was fitted with log_e_ (dose) as a fixed effect and individual subject intercept fitted as random effects. Estimates of the mean slope of log_e_ (dose) were reported along with corresponding 90 % confidence intervals.

To evaluate the accumulation ratio and time invariance of the repeat dose cohorts, a statistical analysis was performed after a log transformation of the data from all active treatment groups. A mixed effect model was fitted with treatment group, day, and treatment group by day interaction as fixed effects and subject as a random effect. Day 14 was compared with Day 1 in order to estimate the accumulation ratio and time invariance ratios for each treatment group. The ratios were calculated by back-transforming the difference between the least squares means. Using the pooled estimate of variance, 90 % confidence intervals were calculated for the difference and then back-transformed.

A mixed effects model was used to analyze the ratio to baseline fractional increase from control CD11b values over time. The model included the same effect as mentioned above except for time (hours). Subject was fitted as a random effect. In the repeat dose cohorts, a mixed effects model was used to analyze the ratio to baseline fractional increase from control CD11b values (treatment group for all pre-treatment data was set to the same dummy value, regardless of the treatment the subject went on to receive). The model included the following fixed effects (effects were fitted as categorical: time (hours) and treatment. Treatment*time and time* baseline interactions were fitted. For each day, a separate mixed model was fitted with time.

Another mixed effects model was used to analyze the weighted mean (0–9 h) ratio to baseline fractional increase from control CD11b values. The model included the following fixed effects (effects were fitted as categorical): day and treatment. Treatment*day interaction was fitted.

In the single and repeat dose cohorts, adjusted geometric means for each treatment and time point were calculated along with 95 % CIs. Point estimates of the treatment differences (each danirixin dose vs. placebo) and their associated 95 % confidence intervals were also calculated (using the pooled estimate of variance) and were back-transformed to provide point estimates and 95 % confidence intervals for the ratios. The ratios were converted into percent values to give the percent inhibition of danirixin versus placebo and 95 % confidence intervals.

A Bayesian analysis was conducted to derive the posterior probability distributions for the ratio to baseline fractional increase from control CD11b values at 24 h. The probabilities of seeing the percent inhibition of danirixin versus placebo greater than 0 %, 50 % and greater than 60 % were derived from the frequentist analysis mixed effects model. A students T cumulative distribution function was used to obtain the probabilistic statements, assuming a non-informative prior.

#### Study 2

The pharmacokinetic parameters AUC (0 to 24 h), AUC (0 to ∞), and Cmax were derived from the blood concentration of study drug. To assess age and gender, the exposures from Cohorts 1 and 2 (both in the fasted state) were combined. Following log_e_-transformation, AUC (0 to ∞) and Cmax of study drug were separately analyzed using a mixed effects model with fixed effect terms age and gender. Age-gender interaction was fitted and explored. To estimate the food effect on the primary pharmacokinetic endpoint, data from Cohort 1 were used. Following log_e_-transformation, AUC (0 to ∞) and Cmax of study drug were separately analyzed using a mixed effects model with fixed effect terms for regimen and period. Subject was treated as a random effect in the model. The same method of analysis, as for the food effect analysis, was applied to estimate the repeat oral doses of omeprazole on the primary pharmacokinetic endpoints, except for the exclusion of period from the model. In the single dose cohorts, a mixed effects model was used to analyse the ratio to baseline fractional increase from control CD11b values. The model included the following fixed effects (effects were fitted as categorical): period (6 levels), time (hours) and treatment. Subject baseline and period baseline were fitted as continuous covariates. Treatment*time and time*period baseline interactions were fitted. Subject was fitted as a random effect.

## Results

### Subject disposition

Thirty subjects were enrolled in the FTIH study and received at least one dose of study drug. One subject withdrew consent and withdrew from the study. Sixteen subjects were enrolled in Cohort 1 of Study 2; one subject was withdrawn after withdrawing consent. Sixteen subjects enrolled in Cohort 2 of Study 2; all subjects completed the study. The demographics and baseline characteristics for these subjects are shown in Tables 1 and 2.

**Table 1:** Demographics and baseline characteristics of study subjects, Study 1 (FTIH)

	Cohorts 1 and 2	Cohorts 3 and 4			Cohort 5	
		Placebo RD	danirixin 50 mg RD	danirixin 200 mg RD	Placebo SD	danirixin 400 mg SD
**Age**, yrs mean (SD)	35 (13)	35 (8)	29 (10)	33 (14)	27 (5)	25 (4)
**n, (% male)**	30 (100 %)	8 (100 %)	10 (100 %)	10 (100 %)	5 (100 %)	10 (100 %)
**Race**, n (%)						
White	25 (83)	6 (75)	10 (100)	9 (90)	4 (80)	7 (70)
African-American	3 (10)	2 (25)	0	0	1 (20)	1 (10)
Asian	2 (6)	0	0	1(10)	0	2 (20)
**BMI** (kg/m^2^) mean (SD)	25 (2)	26 (3)	25 (2)	25 (3)	24 (4)	24 (2)

**Table 2:** Demographics and baseline characteristics of study subjects, study 2 (fed vs. fasted, elderly, omeprazole interaction)

Study 2, Fed vs. Fasted Mean (range)			
	Part A	Part B	
		danirixin	Placebo
Age, years, mean (SD)	51 (8)	70 (2)	70 (6)
Sex (M/F)	8/8	6/6	2/2
Ethnicity, n (%)			
Hispanic/Latino	6 (38)	0	0
Not Hispanic/Latino	10 (63)	12 (100)	4 (100)
Race, n (%)			
African-American	1 (6)	0	0
White	15 (94)	11 (92)	4 (100)
Mixed	0	1 (8)	0
Height (cm), mean (SD)	174 (7)	172 (11)	171 (8)
Weight (kg), mean (SD)	80 (11)	80 (13)	77 (15)
BMI (kg/m^2^), mean (SD)	26 (3)	27 (3)	26 (3)

### Safety and tolerability

There were no serious adverse events and no subjects withdrawn due to adverse events. The most frequent adverse event during the FTIH study, irrespective of causality, was headache, which occurred in 14 subjects (5 subjects on placebo, 1 subject each on 10 mg and 100 mg single dose, 2 subjects each on 200 mg and 400 mg single dose, and 2 subjects on 200 mg repeat dose). Headache that was considered by the investigator to be possibly related to drug treatment occurred in 9 subjects (12 %). Somnolence occurred in five subjects (1 subject on placebo, 1 subject each on 10 mg and 200 mg single dose, and 2 subjects on 100 mg). Hot flushes occurred in four subjects (2 subjects on placebo, 1 subject on 200 mg single dose, and 1 subject on 200 mg repeat dose). Abdominal pain occurred in 4 subjects (1 subject each on 10 mg and 200 mg single dose and 1 subject each receiving 50 mg and 200 mg repeat dose).

In Study 2, 11 subjects reported 12 adverse events (34 %). Four adverse events were reported in subjects receiving danirixin 100 mg in the fasted state (headache, dizziness, nausea, and infusion site hemorrhage). Two adverse events were reported in subjects receiving danirixin 100 mg in the fed state (headache, dyspepsia). Two adverse events were reported in combination with omeprazole (nasopharyngitis, sinusitis). Three adverse events were reported in elderly subjects receiving danirixin 100 mg in the fasted state (application site rash, back pain, and dermatitis atopic) and one adverse event was reported in subjects receiving placebo (viral gastroenteritis). There were no reports of neutropenia or sustained decreases in neutrophil count. However, in the FTIH study, one subject was withdrawn from the study after the reduction in neutrophil levels met protocol stopping criteria of less than 1.5 × 10^9^/L; the neutrophil count was fully reversed upon discontinuation of study drug. This subject was noted to have a low normal neutrophil count at screening (1.9 × 10^9^/L).

### Study 1: FTIH

#### Cohorts 1, 2, and 5 (single dose)

Systemic exposure to single daily doses of danirixin 10 mg, 25 mg, 50 mg, 100 mg (as 2 × 50 mg tablets), and 200 mg increased with increasing dose. The slope log versus log for the (AUC) (0 to ∞ was 1.122 (90 % CI: 0.992, 1.253) and for Cmax was 1.046 (90 % CI: 0.919, 1.172) indicating a proportional increase across the dose range. However, the 100 mg dose (2 × 50 mg) deviated from the dose proportionality shown by the other dose levels and had similar exposure results to 50 mg. The addition of danirixin 400 mg (Cohort 5) in the power model suggested a dose proportional increase in Cmax over the 10–400 mg dose range (1.019; 90 % CI: 0.913, 1.126) but a deviation away from dose proportionality for AUC_(0-∞)_ (1.117; 90 % CI: 1.001, 1.234).

The time at which the maximum blood concentrations were observed (Tmax) was consistent across the dose range studied (median: 1–2 h; range: 1 to 3 h). The apparent terminal half-life was approximately 6 h and was consistent across the dose range studied.

**Fig. 4:**
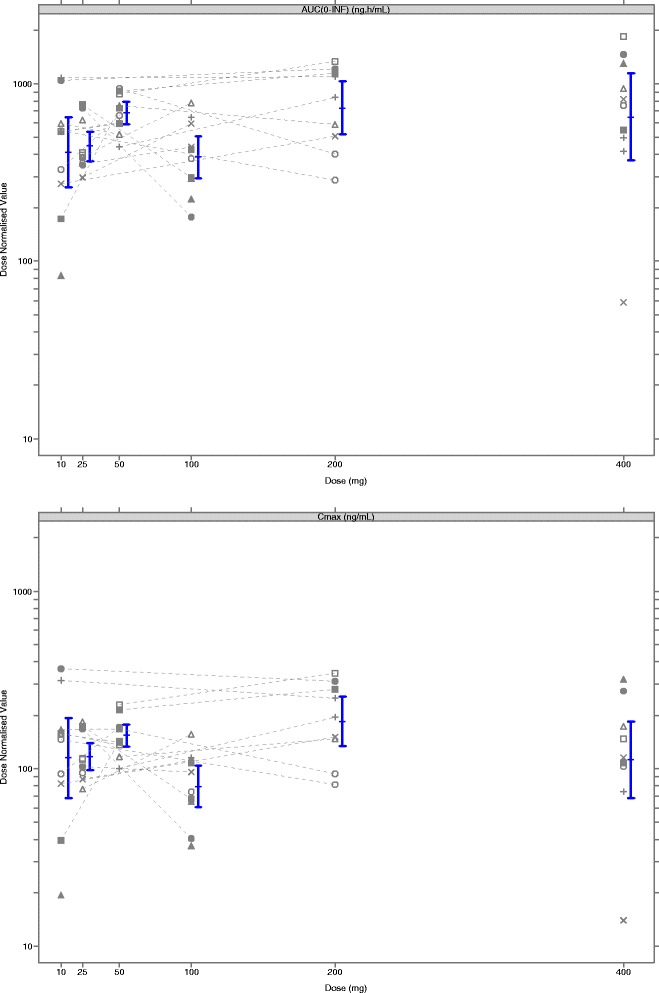
Plot of individual subject danirixin blood dose-normalized AUC (A) and Cmax (B) versus dose for cohorts 1, 2, and 5 in Study 1 (FTIH). The geometric means (with 90 % confidence intervals) are also shown

Figure 4 shows the individual and geometric means of danirixin blood dose-normalized Cmax versus dose using 10 mg as a reference dose. Subjects dosed with 100 mg had a lower Cmax than predicted and four subjects had higher Cmax values than expected (two subjects randomized to 10 mg followed by 200 mg and two subjects randomized to 50 mg followed by 200 mg). These four subjects also had a comparatively high AUC_(0-∞)_. One subject had a high AUC_(0-∞)_ following dosing with danirixin 50 mg but over 2-fold lower AUC_(0-∞)_ than expected following dosing with danirixin 200 mg. Following dosing with danirixin 400 mg, two subjects had higher Cmax than expected and also had higher AUC_(0-∞)_.

#### Cohorts 3 and 4 (14-day repeat dose)

Repeat dosing of danirixin 50 mg and 200 mg resulted in lower exposure on Day 14 than on Day 1. The ratio of adjusted geometric means for AUC _(0-t)_ was 0.846 (90 % CI: 0.553, 1.295) for danirixin 50 mg and 0.989 (90 % CI: 0.619, 1.579) for danirixin 200 mg after repeat dosing. An analysis of AUC and terminal half-life was performed to assess time invariance following repeat dosing with danirixin 50 mg and 200 mg. The AUC exposures were less, on average, on Day 14 compared with Day 1. An average decrease of 21 % (90 % CI: −48 %, 20 %) and 7 % (90 % CI: −42 %, 47 %) was observed for danirixin 50 mg and 200 mg, respectively, indicating that there was no evidence of time invariance. The t½ was greater on average by 33 % (90 % CI:-3 %, 82 %) on Day 14 compared with Day 1 for danirixin 50 mg, suggesting no evidence of time invariance based on t½. However, there was evidence to suggest an increase in t½ on Day 14 compared with Day 1 for danirixin 200 mg as t½, on average, was greater by 71 % (90 % CI: 26 %, 131 %).

### CD11b surface expression on neutrophils

Approximately 50 % inhibition in weighted mean (0–9 h) CXCL1-induced CD11b expression was observed following single doses of danirixin 50 mg (ratio of active to placebo 0.445; 95 % CI: 0.257, 0.772) and 100 mg (0.502; 95 % CI: 0.299, 0.845) and 72 % inhibition (0.282; 95 % CI: 0.161, 0.493) following 200 mg compared with placebo. The ratio to baseline fractional increase from control CD11b expression at 24 h for single doses of danirixin 50 mg, 100 mg (2 × 50 mg), and 200 mg was 1.908 (95 % CI: 0.853, 4.265), 1.326 (95 % CI: 0.565, 3.110), and 0.516 (95 % CI: 0.212, 1.255), respectively. Bayesian analyses revealed that the probability of danirixin inhibiting CXCL1-induced CD11b expression relative to placebo at 24 h postdose at single doses of 50 mg, 100 mg, and 200 mg was 0.057, 0.26, and 0.93, respectively.

Repeat doses of danirixin 50 mg demonstrated an average of 67 % and 70 % weighted mean (0 to 9 h) CXCL1-induced CD11b inhibition on day 1 (0.335; 95 % CI: 0.227, 0.495) and day 14 (0.399; 95 % CI: 0.258, 0.617), respectively, relative to placebo. Repeat doses of danirixin 200 mg demonstrated an average of 70 % and 50 % weighted mean (0 to 9 h) CXCL1-induced CD11b inhibition on day 1 (0.301; 95 % CI: 0.209, 0.435) and day 14 (0.501; 95 % CI: 0.319, 0.788), respectively, relative to placebo. Bayesian analyses revealed that the probability of repeat doses of danirixin 200 mg inhibiting CXCL1-induced CD11b expression relative to placebo was 1.0.

**Fig. 5:**
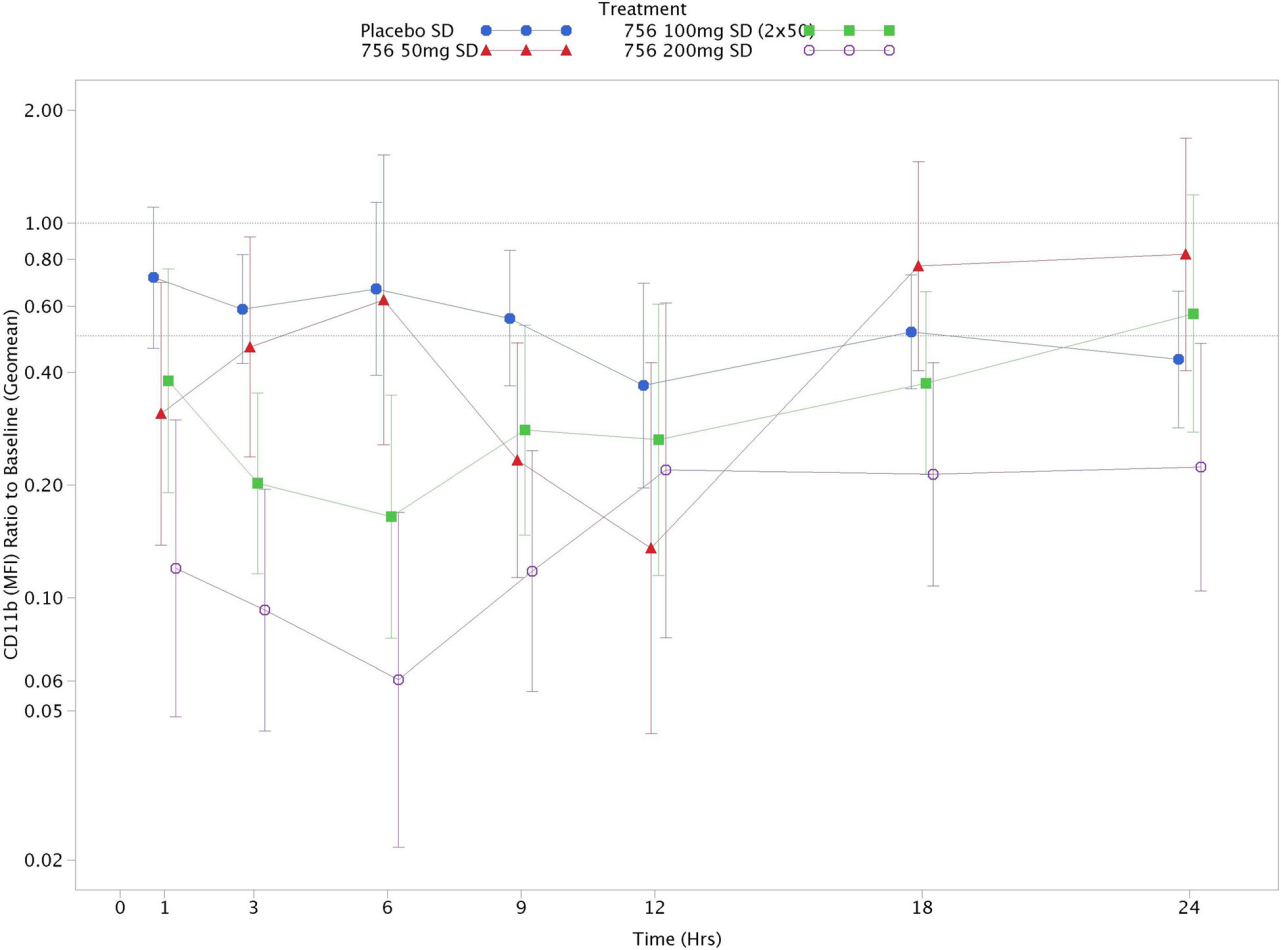
Adjusted geometric means of ratio to baseline ex vivo CXCL-induced CD11b expression (0–24 h) versus time for single dose Study 1 (FTIH): fractional increase from control (CXCL1 0 nM) with 95 % CIs

**Fig. 6:**
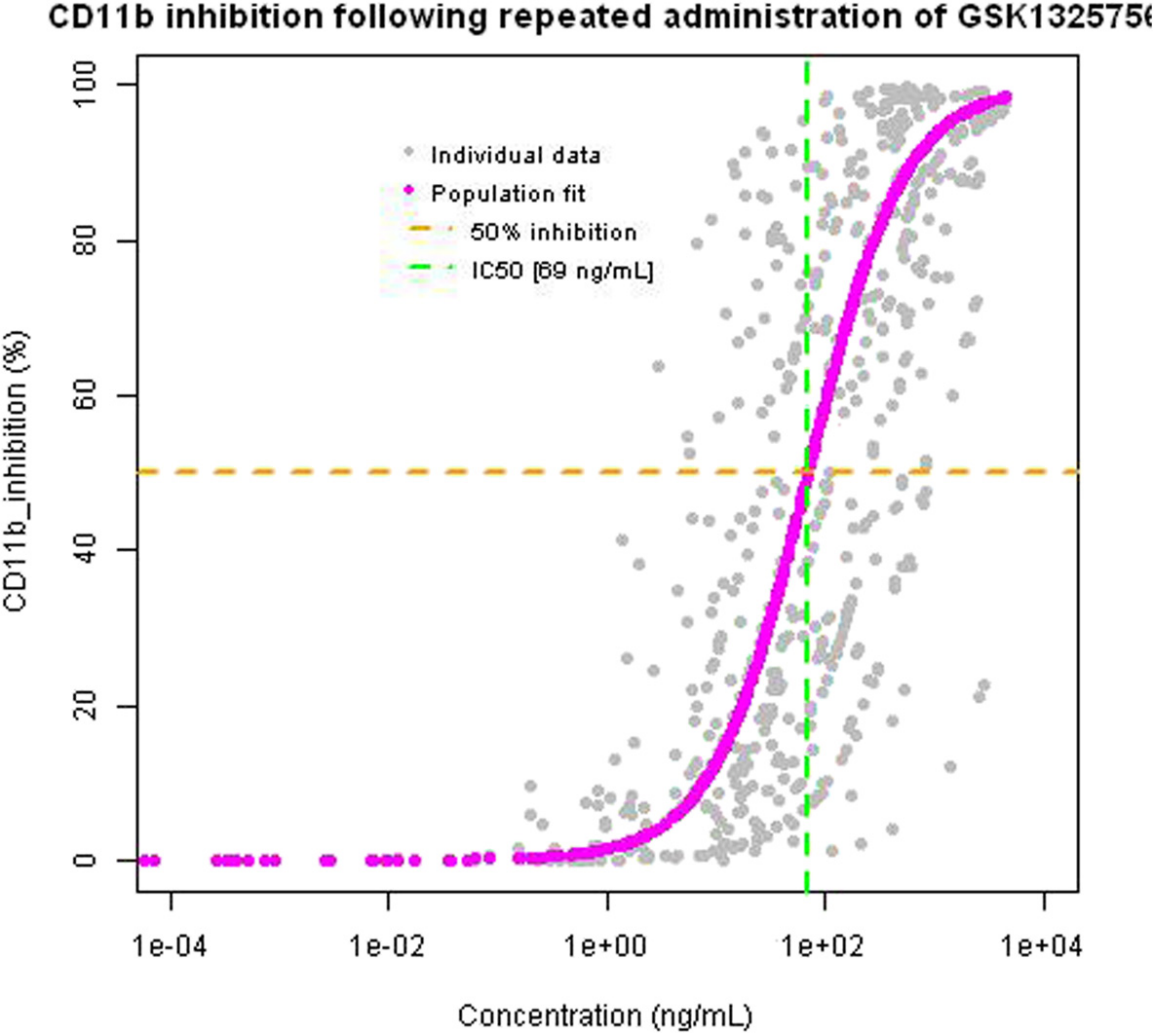
Plot of inhibition of ex vivo CXCL1-induced CD11b expression versus whole blood concentration of danirixin for Study 1. The plot shows all timepoints where results are available for both danirixin whole blood concentration and ex vivo CXCL1-induced CD11b expression

In general, repeat doses of danirixin 50 mg and 200 mg demonstrated an average of 50 % inhibition in CXCL1-induced CD11b expression at various time points over the 24 h post dose on Day 1 (Fig. 5) and for most time points on Day 14, though high variability was observed across all treatments. The ratio to baseline fractional increase from control CD11b expression at 24 h for repeat doses of danirixin 50 mg on Day 1 was 0.374 (95 % CI: 0.132, 1.057) and on Day 14 was 0.403 (95 % CI: 0.226, 0.718). The ratio to baseline fractional increase from control CD11b expression at 24 h for repeat doses of danirixin 200 mg on Day 1 was 0.303 (95 % CI: 0.107, 0.861) and Day 14 was 0.348 (95 % CI: 0.192, 0.631). Fig. 6 shows the concentration-response plot for the effect of danirixin on the CXCL1-induced CD11b expression (shown in terms of percent inhibition), and clearly shows that a direct relationship exists between blood concentrations of danirixin and reduction in CXCL1-induced CD11b expression. Population PK/PD modeling of the data from the FTIH study suggests that the maximum possible inhibition of CXCL1-induced CD11b expression could be achieved and that the IC_50_ for this inhibition was 69 ng/mL (95 % CI: 17.6 to 120 ng/mL).

### Study 2: Fed/fasted, omeprazole interaction and elderly pharmacokinetics

**Fig. 7:**
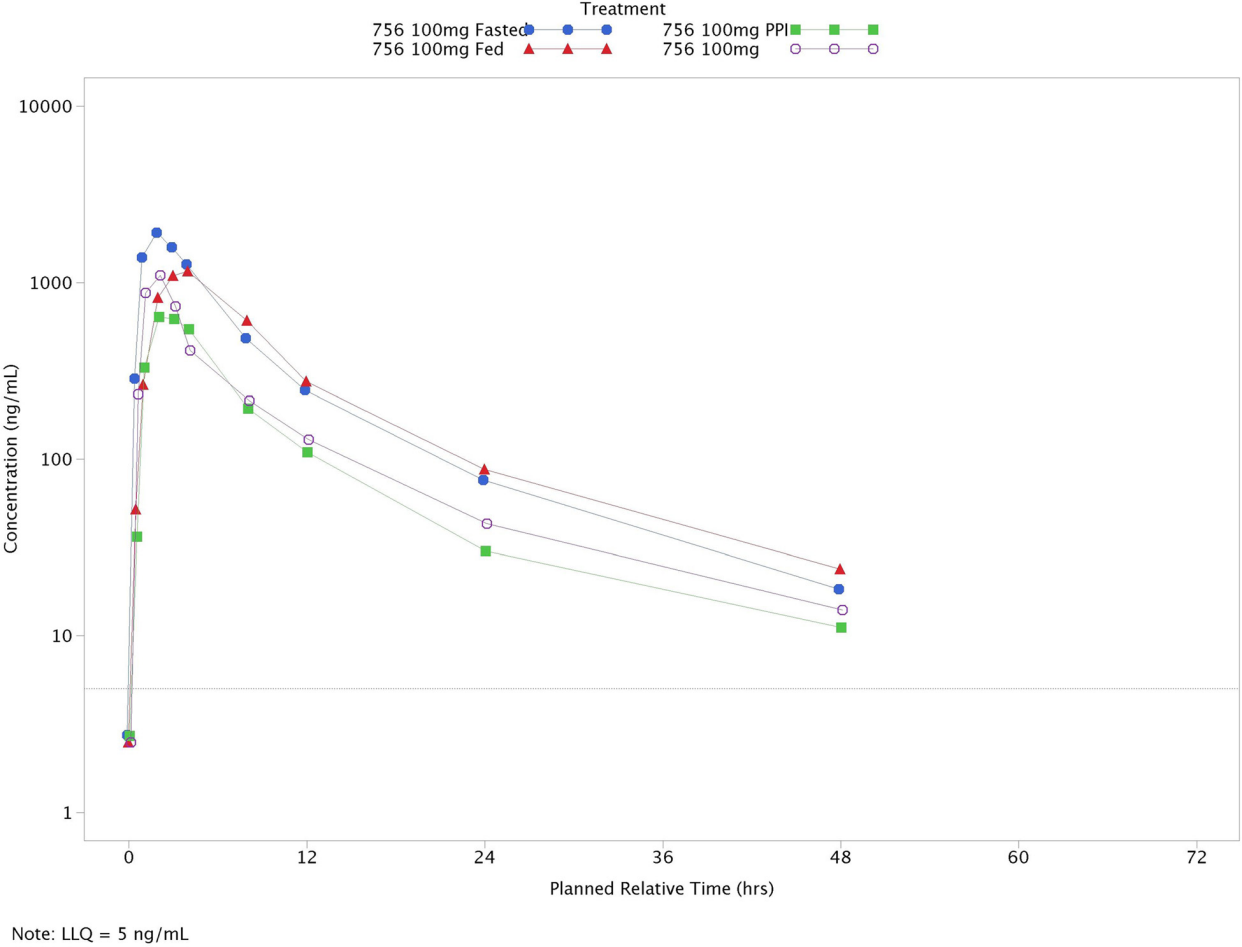
Mean pharmacokinetics concentration plot versus time for Study 2 (Fed versus Fasted, Elderly, Omeprazole Interaction)

There was a statistically significant reduction in Cmax of 37 % (90 % CI: 24 % to 48 %) when danirixin 100 mg was administered following food (Table 3 and Fig. 7). The average decrease of 16 % in AUC_(0-∞)_ following administration in the presence of food was not statistically significant (90 % CI: −3.1 % to 32 %). The median Tmax was delayed in the presence of food compared with that in fasted subjects (3 h and 2 h, respectively). There was some evidence of a period effect, with lower average Cmax and less variability in Cmax in Period 2. There was also some evidence of a treatment-period interaction. Subjects randomized to fasted followed by fed had lower average Cmax and AUC_(0-∞)_ in the fed period, while subjects on the other treatment sequence had higher average Cmax and AUC_(0-∞)_ in the fed period. Subjects with the highest exposure in the danirixin 100 mg fasted period did not also have the highest exposure in the danirixin 100 mg fed period.

**Table 3:** Summary of statistical analysis of fed/fasted comparison (Study 2)

	Danirixin 100 mg Fed		Danirixin 100 mg Fasted		Treatment Difference(Fed vs. Fasted)	
	n	Adjusted Geometric Mean(SE Logs)	n	Adjusted Geometric Mean(SE Logs)	Estimate	90 % CI
**AUC** (0-∞)(ng h/mL)	15	12,635 (0.082)	13	15,047 (0.088)	0.840	(0.684, 1.031)
**Cmax**(ng/mL)	16	1411 (0.076)	15	2239 (0.078)	0.630	(0.523, 0.758)

There was a reduction in exposure when danirixin was administered following dosing with omeprazole 40 mg once daily for 5 days (Fig. 5). The decreases of 56 % for AUC_(0-∞)_ (90 % CI: 41 % to 66 %) and 64 % for Cmax (90 % CI: 50 % to 73 %) were statistically significant, while the median Tmax was unchanged at 2 h for both treatments (Table 4).

**Table 4:** Summary of statistical analysis of proton pump inhibitor comparison (Study 2)

	Danirixin 100 mg Fasted + PPI		Danirixin 100 mg Fasted		Treatment Difference (Fasted + PPI vs. Fasted)	
	n	Adjusted Geometric Mean (SE Logs)	n	Adjusted Geometric Mean (SE Logs)	Estimate	90 % CI
**AUC** (0-∞)(ng h/mL)	12	6220 (0.1705)	13	14,020 (0.1659)	0.444	(0.337, 0.584)
**Cmax **(ng/mL)	15	813 (0.1646)	15	2235 (0.1646)	0.364	(0.267, 0.496)

In elderly subjects receiving danirixin 100 mg, the AUC_(0-∞)_ and Cmax were approximately half that observed for subjects aged 40 to 64 years (AUC_[0-∞]_, 90 % CI: 14 % to 73 %; Cmax, 90 % CI: −2.5 % to 73 %). In addition, the median Tmax for the fasted elderly cohort was earlier than that of the younger fasted cohort (1.5 h compared to 2 h).

Female subjects had approximately 1.5-fold higher average exposures compared to males (AUC_[0-∞]_, 90 % CI: 0.826 to 2.604; Cmax, 90 % CI: 0.806 to 3.064). However, due to the wide variability, these apparent differences between the sexes were not statistically significant. An exploratory analysis suggested that the absorption of danirixin was higher and that the clearance was lower in female subjects compared to male subjects. There was no evidence of an age-gender interaction; however, as the data are highly variable and the populations were imbalanced in terms of male and female age ranges, further work is needed to adequately define the importance of age and gender on the absorption of danirixin.

## Discussion

The data presented in this report are from the first two clinical studies with danirixin in humans to assess the initial safety, tolerability, pharmacokinetics and pharmacodynamics of single and repeated doses under conditions relevant to the target patient population. Administration of danirixin was generally well-tolerated at single doses up to 400 mg and following once daily dosing for 14 days at doses of 50 mg and 200 mg. Importantly, subjects did not develop low neutrophil counts or overt neutropenia, as has been reported with other CXCR2 antagonists [6–8]. Danirixin demonstrated a dose-dependent inhibition of CXCL1-induced CD11b expression following single doses between 50 to 200 mg, which was maintained after 14 days of dosing. The optimal extent of pharmacodynamic activity needed is unknown, but it is likely that less than complete inhibition of CXCR2 activity is desirable in order to obtain a balance between clinical benefit, while minimizing the impact on innate immunity and host defense. Although the magnitude and duration of CXCR2 antagonism needed to achieve a pharmacologic and clinical effect in disease are unknown the pharmacokinetic data suggest that twice-daily dosing with danirixin may be needed to maintain blood concentrations at an appropriate level.

The prevalence of gastroesophageal reflux disease (GERD) and the use of gastric acid suppressing agents in COPD, one of the disease targets for a CXCR antagonist, is high [11–13]. Proton pump inhibitors have been reported to increase intragastric pH above 4.0 for approximately 50 % of the time over a 24 h interval [14]. Acidic conditions are required for optimal dissolution of danirixin. The formulation strategy for danirixin has thus far been focused on optimizing the disintegration and dissolution of the product in the stomach, which would then be expected to deliver a solution of the drug for absorption to the duodenal region of the small intestine. An intra-gastric pH at a steady state above 4.0 during a significant proportion of the day has potential implications for the solubility and absorption of danirixin. Statistically significant reductions in danirixin AUC and Cmax were observed in the presence of omeprazole.

The effect of omeprazole on the bioavailability of danirixin suggests an important drug interaction requiring further evaluation to determine appropriate dosing recommendations in patients using gastric acid reducing agents. Additional drug interactions that may impact danirixin have not been evaluated. However, one study has assessed the potential for metabolic drug interactions. In this study a bile sample was obtained using a non-invasive sampling technique [15]. The results demonstrated that glucuronide conjugation is likely to be the major route of metabolism for danirixin. Oxidative metabolism contributed only in a minor way indicating that cytochrome P450-mediated drug interactions are not likely to be a concern for danirixin.

In the presence of a high fat meal, the AUC and Cmax data suggest that the rate, but not the extent of absorption of danirixin was reduced, as would be expected from a molecule with this solubility profile. Furthermore, these data also suggest that in the fasted state the average exposure to danirixin in the elderly was half that of younger subjects, as reflected by lower AUC and Cmax measurements. In addition, in the fasted state, very high inter-subject variability in the exposure measurement was observed in elderly subjects. Administration of danirixin with food resulted in reduced variability in a range that is considered acceptable.

## Conclusions

The data in the reported studies indicate that danirixin was well-tolerated, with a favorable pharmacokinetic and pharmacodynamic profile in healthy adult subjects, including elderly subjects. The dose-dependent inhibition of CXCL1-induced CD11b expression following single doses between 50 to 200 mg suggests the potential for clinical benefit in disease indications where an excessive neutrophil response has an important role in pathogenesis. The co-administration of daily omeprazole and use in the elderly will have to be taken into consideration when progressing to evaluation in patient populations.
